# Intermittent induction of LEA peptide by lactose enhances the expression of insecticidal proteins in *Bacillus thuringiensis*

**DOI:** 10.1002/2211-5463.13448

**Published:** 2022-06-15

**Authors:** Mahmuda Akthar, Tomoko Shimokawa, Yinghan Wu, Taichi Arita, Kazuhiro Mizuta, Yuria Isono, Minoru Maeda, Shinya Ikeno

**Affiliations:** ^1^ Department of Biological Functions Engineering, Graduate School of Life Science and Systems Engineering Kyushu Institute of Technology, Kitakyushu Science and Research Park Kitakyushu, Fukuoka Japan; ^2^ Bioindustry Division Kyushu Medical Co, LTD Fukuoka Japan

**Keywords:** *Bacillus thuringiensis*, crystal protein, intermittent induction, lactose, late embryogenesis abundant (LEA) peptide

## Abstract

Cry toxins from *Bacillus thuringiensis* (Bt) have been extensively applied in agriculture to substitute the use of chemical insecticides. We have previously reported the use of a coexpression system in which late embryogenesis abundant (LEA) peptides under the control of the *lac* promoter increase the expression of insecticidal proteins in Bt. The use of lactose to induce the expression of LEA peptides may be a desirable alternative to isopropyl β‐D‐thiogalactopyranoside, the most frequently used inducer for recombinant protein expression. In this study we investigated the use of lactose as an inducer for optimal protein expression. We observed enhanced insecticidal Cry protein expression by applying a simple technique based on intermittent induction, and then optimized concentration and the point of induction time from the 11^th^ h to the 15^th^ h. Our data suggest that intermittent induction of lactose might be a new technique for the enhancement of bacterial protein expression.

## Graphical abstract

The late embryogenesis abundant peptide coexpression method, in which protein expression is made more efficient by coexpressing a short peptide consisting of 13 amino acids, was used to increase the expression of insecticidal proteins expressed in *Bacillus thuringiensis*. Late embryogenesis abundant peptide expression was induced by intermittent addition of lactose as an expression inducer to the culture medium, resulting in effective promotion of Cry protein expression.

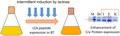

**Abbreviations**
Bt: *Bacillus thuringiensis*
*E. coli*
: *Eschericia coli*
IPTG: isopropyl β‐D‐thiogalactopyranoside
LEA: late embryogenesis abundant
SDS/PAGE: sodium dodecyl sulfate/polyacrylamide gel electrophoresis
TSB: tryptic soy broth

Cry toxins from *Bacillus thuringiensis* (Bt) have been extensively applied in agriculture to substitute the use of chemical insecticides [1]. Globally, mosquitos, particularly *Aedes aegypti*, are considered one of the most dangerous vectors, causing diseases like malaria, yellow fever, dengue, and chikungunya in tropical areas [2]. Bt can restrict the defoliators and mosquitoes, reducing the use of chemical insecticides [3]. The main attractions of using Bt to restrict mosquito and black fly populations are its high insecticidal activity, absence of resistant insect populations, nontoxicity to nontarget organisms, and the growth of chemical insecticide‐resistant insect populations [4]. However, due to the lucrative market, most R&D companies still prioritize chemical insecticides.

*Escherichia coli* remains the host of choice for recombinant expression due to its easy culturing, good product yield, and low cost. To this effect, the lac operon and its control elements are used most frequently [5, 6, 7, 8].

Our group has attempted to increase the expression level of insecticidal proteins by using Bt as an expression host and the LEA peptide coexpression method [9]. In this method, LEA peptides are expressed in Bt under the control of the *lac* promoter, increasing the insecticidal protein expression.

However, the lac system has the following limitations: (a) Continuous monitoring is needed for the induction at the optimal cell density. The point of induction may vary across recombinant proteins, making it difficult to automate the process, especially when multiple proteins are expressed side by side (e.g. for a screen); (b) Narrow range of technical issues; (c) From the commercial viewpoint, less appropriate; (d) Toxicity (particularly in the case of human therapeutic protein production), and (e) Expensive [10]. Normally, the addition of isopropyl β‐d‐thiogalactopyranoside (IPTG) induces the expression system with the help of the T7 system [11, 12]. IPTG has a high metabolic burden on the organism and the formation of inclusion bodies [13, 14, 15, 16]. To produce bio‐based insecticides, it is essential to select a proper induction method to gain the optimum result.

Many induction methods have already been established to optimize protein expression with induction time [17, 18, 19, 20]. Induction systems can be induced by adding lactose or any other synthetic inducer to the bacterial growth media for the expression of the recombinant protein. Recently, autoinduction for T7*lac* promoter has been used without observing the cell growth means expression at different cell densities for lactose‐inducible bacterial expression systems [21]. Thus, the LEA peptide coexpression system with intermittent induction by lactose can act like an autoinduction system. The presence of glucose represses T7 RNA polymerase expression, as it is the favored carbon source. Lactose can drive the synthesis of T7 RNA polymerase by aiding the transcription of the target mRNA of the gene cloned in the plasmid vector in the absence of glucose.

Lactose is a more economical compound than glucose and IPTG, and an induction strategy based on lactose alone is appealing [22]. Permease (*lacY*) is important for the induction of lactose and *lacZ* as it imports lactose, which is then converted by β‐galactosidase into allolactose―the molecule that represses the function of the lac repressor. Usually, during induction, the initial stage of the growth phase in autoinduction media is followed by a normal growth rate before glucose is depleted, such that, during the induction period, most of the cells are already in a functional state.

Another good reason to use lactose in autoinduction is that induction starts only after the total available glucose is depleted [23]. The inducing criteria of lactose differ markedly for various kinds of recombinant protein expression compared with IPTG where optimum induction conditions are already established [24]. Despite the advantages of using lactose as an inducer of recombinant protein expression, the system needs to be optimized with respect to the induction parameters and timing [25]. We used the LEA peptide coexpression system induced by lactose to enhance Cry protein expression. This approach achieves the targeted protein expression at a lower cost in an industrial setting compared with the more commonly used IPTG. The use of Bt‐LEA transformant as a host for the expression of recombinant protein coexpressed with LEA peptide using lactose as an inducer would make the production of bio‐based insecticidal proteins more feasible. This research assesses the conditions needed for the efficient expression of Cry protein using an inducible Bt‐LEA peptide coexpression system to determine whether lactose can be used as a commercial inducer. We determined the optimum culture conditions, dosage concentration, timing, and induction pattern.

## Methods

### Strains and expression vector

The strains and the protocol for the construction of the expression vector are detailed in our previous article [9].

### Designing the LEA peptide

To construct different types of LEA peptides such as LEA‐I, LEA‐K, and LEA‐E, we used the Bt codon frequency table to obtain the amino acid sequences. The designed LEA peptides have the following sequences: MDAKDGTKEKAGE, MDAKDKTKEKAKE, and MDAKDETKEKAEE. The synthetic DNAs were acquired from Eurofins Genomics Inc. (Tokyo, Japan) by specifying the following oligonucleotide sequences (Table 1).

**Table 1:** DNA sequences for construction of each LEA gene.

Name	DNA sequence
S‐LEA‐I	5′‐GATCCATGGATGCAAAAGATGGAACAAAAGAAAAAGCAGGTGAATAAT‐3′
AS‐LEA‐I	5′‐CTAGATTATTCACCTGCTTTTTCTTTTGTTCCATCTTTTGCATCCATG‐3′
S‐LEA‐K	5′‐GATCCATGGATGCAAAAGATAAAACAAAAGAAAAAGCAAAAGAATAAT‐3′
AS‐LEA‐K	5′‐CTAGATTATTCTTTTGCTTTTTCTTTTGTTTTATCTTTTGCATCCATG‐3′
S‐LEA‐E	5′‐GATCCATGGATGCAAAAGATGAAACAAAAGAAAAAGCAGAAGAATAAT‐3′
S‐LEA‐E	5′‐CTAGATTATTCTTCTGCTTTTTCTTTTGTTTCATCTTTTGCATCCATG‐3′

### Insertion of the LEA peptide gene into the pHT01 vector

The protocol for the insertion of the LEA peptide gene into the pHT01 vector has been detailed in our previous article [9].

### Development of Bt‐LEA transformants

Three different types of Bt‐LEA transformants: Bt‐LEA‐I, Bt‐LEA‐E, and Bt‐LEA‐K, were used as host cells. The construction process of Bt‐LEA‐I and Bt‐LEA‐K have been detailed in our previous article [9]. A similar protocol was followed to construct Bt‐LEA‐E.

### Culturing and induction of LEA peptide by lactose

We cultured all Bt‐LEA transformants (Bt‐LEA‐I, Bt‐LEA‐E, and Bt‐LEA‐K) by modifying parameters like concentration, induction pattern, and induction timing of lactose as an expression inducer, and determined the protein expression levels in each condition at different timepoints. The entire process was regulated in baffled flasks containing tryptic soy broth (TSB) supplemented with the appropriate antibiotic (chloramphenicol, 5 μg·mL^−1^) and 100× spore‐forming solution at 27 °C and 150 r.p.m. for 48 h. Antibiotic (chloramphenicol, 5 μg·mL^−1^) was omitted for the wildtype culture (nontransformant). To determine the effect of lactose as an inducer, the cultures were induced with 0.0, 0.1, 0.2, 0.5, and 0.1 mmol·L^−1^ IPTG (for control) for different periods of cultivation in accordance with the requirement of each experiment. The induction time was attributed in two different slots: 11^th^, 12^th^, 13^th^, 14^th^, and 15^th^ h, and 16^th^, 17^th^, 18^th^, 19^th^, and 20^th^ h to determine the time of induction. Samples were harvested according to the time after the induction for analysis. The bacterial cell concentration was quantified by measuring the optical density (OD) with the help of a spectrophotometer (ASV11D, AS ONE, Osaka, Japan) at 600 nm. The expression level of the Cry protein was quantified using SDS/PAGE. After confirming the expression level, we compared the induction patterns like intermittent induction (using 10 μL lactose) or all‐at‐once induction (using 50 μL lactose) at the 11^th^, 12^th^, 13^th^, 14^th^, and 15^th^ h for each type of LEA transformant, keeping all the other parameters constant. During this investigation, the samples were also harvested from the 0–12^th^ h at 2 h intervals and thereafter at the 24^th^, 30^th^, 36^th^, and 48^th^ h to observe the cell growth after induction of lactose. After the 48^th^ h of incubation, the Cry protein was harvested from the cell lysate solution. Based on the amount of protein observed on SDS/PAGE for each sample, the optimum conditions for the expression of the insecticidal protein were determined.

### SDS/PAGE analysis

After 48 h of incubation, for each investigation, the sample was collected after measuring the OD at 600 nm, 1 mL of culture was taken, and the cells were harvested by centrifugation at 13 000 *g* for 5 min. After centrifugation, the supernatant was removed, and the pellet was dissolved in 200 μL of distilled water. To prepare the final sample, the solubilized sample was mixed with 2× SDS sample lysis buffer (2× containing 125 mmol·L^−1^ Tris–HCl (pH 6.8), 4% SDS, 20% glycerol, 0.1 mg·mL^−1^ of bromophenol blue, 10% 2‐sulfanylethanol, and 2‐mercaptoethanol) followed by heat‐shock and ice incubation at 95 °C and 5 min. Lastly, 5 μL of each processed sample and Protein Molecular Weight Marker Broad (Takara Bio, Kusatsu, Japan) were loaded into the wells of a gradient polyacrylamide gel (gel concentration: 5–20%, ATTO, Tokyo, Japan) for 30 min at 10.5 mA·gel^−1^. The separated proteins were observed by staining with Coomassie Brilliant Blue (EzStain Aqua, ATTO) and de‐staining after 2 h. imagej (NIH, Bethesda, MD, USA) was used to evaluate the results of SDS/PAGE [26]. The marker acted as a reference to quantify the average density of the nontransformant bands (Control 1) and the 66.4 kDa band. The 66.4 kDa protein was considered the insecticidal protein Cry11Aa after comparing it to the nontransformant band [27].

### Determination of glucose and lactose in the medium and cell

To determine the glucose and lactose concentration in the medium and the cells, the glucose, and lactose detection kit was purchased from Boehringer Mannheim/R‐Biopharm (Darmstadt, Germany). The highest expression level was confirmed in the case of the Bt‐LEA‐E transformant with a concentration of 0.1 mmol·L^−1^ from SDS/PAGE analysis. So, the concentration of glucose, lactose, and galactose was only detected in the case of Bt‐LEA‐E transformation with the treatment of intermittent induction (10 μL of 0.1 mol·L^−1^) and all at once induction (50 μL of 0.1 mol·L^−1^) at the incubation time of 10^th^, 11^th^, 12^th^, 13^th^, 14^th^, 15^th^,16^th^, and 24^th^ h. We started the induction at the 11^th^ h exactly both in the pattern of intermittent (10 μL) and all at once induction (50 μL). To obtain accurate results, we also checked the concentration of glucose, lactose, and galactose in the case of Bt‐LEA‐E without induction, as the blank, was deducted during the calculation.

## Results and discussion

### Design and construction of recombinant Bt. D142 Strain expressing LEA peptide

From our previous study [9], the Bt‐LEA‐K transformant had a small effect on increasing expression, unlike our previous studies using *E. coli*. Therefore, we designed LEA‐E, which has substituted acidic amino acids instead of basic ones (Table 2). Using the codon frequency table of Bt, we designed Bt‐LEA‐E transformants by adding point mutations to the LEA‐I sequence to scrutinize whether LEA peptide has any effect on the expression of Cry protein of Bt. D142 or not. Point mutations, size, and structure of amino acids affect the function of LEA peptides. From our previous study [28], it is already established that point mutations play a vital role in the enhancement of the function of LEA peptides in the coexpression of targeted proteins in cells. In the amino acid sequence of the 13‐mer LEA peptides, glycine was replaced with glutamic acid at the position of amino acids 6 and 12 in the LEA‐E peptide, and glycine was replaced with lysine at the position of amino acids 6 and 12 in the LEA‐K peptide.

**Table 2:** Amino acid sequences of the mutated LEA peptides.

Peptides	1	2	3	4	5	6	7	8	9	10	11	12	13
LEA‐I	M	D	A	K	D	G	**T**	K	E	K	A	G	E
LEA‐K	M	D	A	K	D	**K** ^a^	T	K	E	K	A	**K** ^a^	E
LEA‐E	M	D	A	K	D	**E**	T	K	E	K	A	**E**	E

^a^ The highlighted bold letters indicate the point mutation in each LEA peptide.

### Expression of cry protein in Bt‐LEA transformants and its effect on cell growth

**Fig. 1:**
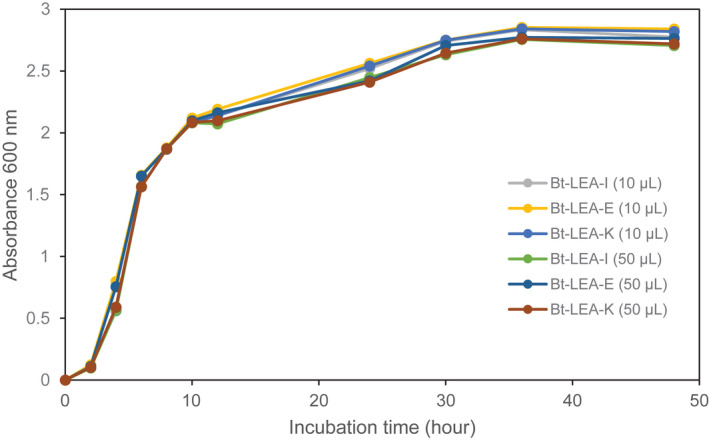
Cell growth curves of recombinant Bt expressing the three mutated LEA peptides showing time dependence in intermittent (10 μL) and all‐at‐once (50 μL) induction of lactose. Induction time of intermittent (10 μL) was followed at the 11^th^, 12^th^, 13^th^, 14^th^, and 15^th^ h, and all‐at‐once (50 μL) induction of lactose at the 11^th^ h. [Colour figure can be viewed at wileyonlinelibrary.com]

One of our major challenges while performing this experiment was to introduce the genes into cells without affecting the expression of endogenous proteins, particularly those for the expression of Cry protein and proliferation of cells. For this, the expression level of Cry protein in the Bt‐LEA transformants (Bt‐LEA‐II) was determined by providing the same conditions as those in the case of nontransformants (Wt, Bt. D142, Fig. 1). The results have already been described in our previous study [9], where the cell growth pattern of the transformants and nontransformants was mostly similar.

### Cell growth and expression of the cry protein in the Bt‐LEA transformants using lactose, intermittently and all at once for induction

The cell growth in the Bt‐LEA transformants was compared between cultures given lactose intermittently (5 × 10 μL) and all at once (50 μL) for induction of gene expression. No drastic change in expression was observed between the three Bt‐LEA transformants (Bt‐LEA‐I, Bt‐LEA‐E, and Bt‐LEA‐K). However, the highest enhancement was observed in the case of Bt‐LEA‐E transformants (Fig. 1) by the intermittent induction of lactose. In the LEA peptide coexpression system, this enhancement might be due to the LEA peptide acting either as a molecular chaperon or exerting a molecular shield effect on the cell. In Bt‐LEA‐E transformant, the negatively charged (acidic) amino acids, their polarity, hydrophilicity, and position (6^th^ and 12^th^ position) can act as key indicators for this efficient expression. In the LEA peptide coexpression, the importance of the size and position of amino acids that play a vital role in the coexpression system have been already reported by Pathak *et al*. [28]. Finally, the most important feature of the Bt‐LEA‐E transformant is its induction by intermittent induction of lactose.

### Effect of induction time on efficient expression of cry proteins

SDS/PAGE data were analyzed by imagej, which is the most commonly used method to check protein expression levels where the expression level of the Bt Cry protein (Cry11Aa) was confirmed in conjugate with the LEA peptide coexpression system. The Bt‐LEA transformants LEA‐I, LEA‐K, and LEA‐E and nontransformants (Wt) showed the protein band at 66.4 kDa. The band intensity of the Bt‐LEA‐I and Bt‐LEA‐K was more pronounced than the band intensity of the Wt‐ and IPTG‐induced expression vectors. To determine the optimum conditions for the expression of the Cry protein, we varied lactose concentrations and induction times from 11^th^–15^th^ (Table 3) and 16^th^–20^th^ h (Table 4). Cry protein expression was induced with 0.0, 0.1, 0.2, and 0.5 mmol·L^−1^ of lactose and 0.1 mmol·L^−1^ of IPTG for LEA‐I, LEA‐E, and LEA‐K. Induction timing and the concentration of lactose were considered to check the intensity of expression. Expression of the Cry protein was the highest at 0.1 mmol·L^−1^ concentration of lactose during the 11^th^–15^th^ h in the case of LEA‐E. Lactose was more effective than IPTG in the LEA peptide coexpression system, particularly for the 0.1 mmol·L^−1^ concentration of both lactose and IPTG in the case of the Bt‐LEA‐E transformant. These results indicate that the expression level of the Cry protein was attributed to the coexpression of the LEA‐I, LEA‐E, and LEA‐K peptides by the LEA peptide coexpression system. For efficient expression of the Cry protein, the induction time was considered to be relevant for Bt. D142 as the logarithmic phase ends at 10^th^ h after the initiation of the culture, leading to the stationary phase. The continuous addition of lactose has an influence on the Cry protein expression in this coexpression system. Lactose, as an inducer, was more effective in comparison to IPTG for the enhancement of expression in *Helicobacter pylori*, where the recombinant genes were able to express the targeted proteins [29]. In this investigation, the dosages and inducing times were 0.8, 50 g·L^−1^ and 4 h for rHpa A; 0.8, 100 g·L^−1^ and 4 h for rLTKA63; and 1.2, 100 g·L^−1^ and 5 h for both rUreB and rLTB [30].

**Table 3:** SDS/PAGE analysis of insecticidal protein expression in transformant Bt by lactose monohydrate and IPTG induction time at the 11^th^, 12^th^, 13^th^, 14^th^, and 15^th^ h‐each h 10 μL.

Bt	0.0 mmol·L^−1^			0.1 mmol·L^−1^			0.2 mmol·L^−1^			0.5 mmol·L^−1^			0.1 mmol·L^−1^ (IPTG)		
	LEA‐I	LEA‐E	LEA‐K	LEA‐I	LEA‐E	LEA‐K	LEA‐I	LEA‐E	LEA‐K	LEA‐I	LEA‐E	LEA‐K	LEA‐I	LEA‐E	LEA‐K
1.00	0.21	2.10	0.85	2.56	2.97	0.67	2.41	1.94	1.19	2.87	2.45	0.85	0.96	1.32	0.72

**Table 4:** SDS/PAGE analysis of insecticidal protein expression in transformant Bt by lactose monohydrate and IPTG induction time at the 16^th^, 17^th^, 18^th^, 19^th^, and 20^th^ h‐each h 10 μL.

Bt	0.0 mmol·L^−1^			0.1 mmol·L^−1^			0.2 mmol·L^−1^			0.5 mmol·L^−1^			0.1 mmol·L^−1^ (IPTG)		
	LEA‐I	LEA‐E	LEA‐K	LEA‐I	LEA‐E	LEA‐K	LEA‐I	LEA‐E	LEA‐K	LEA‐I	LEA‐E	LEA‐K	LEA‐I	LEA‐E	LEA‐K
1.00	1.19	1.17	1.26	1.22	2.36	1.32	1.56	1.48	1.61	1.55	1.49	1.71	1.28	1.50	1.32

We used intermittent and all‐at‐once induction. In another approach by Tian *et al*. [31], the induction of lactose for the optimization of fermentation resulted in 1382 g of cell mass, representing 84% enrichment in cell mass in comparison to that obtained from IPTG induction.

### Optimal inducing concentrations and induction pattern of lactose

In this investigation, lactose with two different concentrations and induction patterns was used to determine the optimum conditions for Cry protein expression. In addition, only lactose was using either as an inducer or a carbon source for the growth of bacteria to express the Cry protein. We can infer that the intermittent induction pattern with 10 μL of lactose might be one of the crucial factors for the enhancement of Cry protein expression in comparison to IPTG. This intermittent induction using the lactose phenomenon was not formerly illustrated in the expression of the protein in association with the LEA peptide coexpression system, particularly for the Cry protein expression. Thus, this may be why the intermittent induction works better in comparison to the all‐at‐once induction as the bacterial cell can completely utilize lactose as glucose depletes with time, because the presence of glucose often prevents the use of secondary carbon sources [32].

**Fig. 2:**
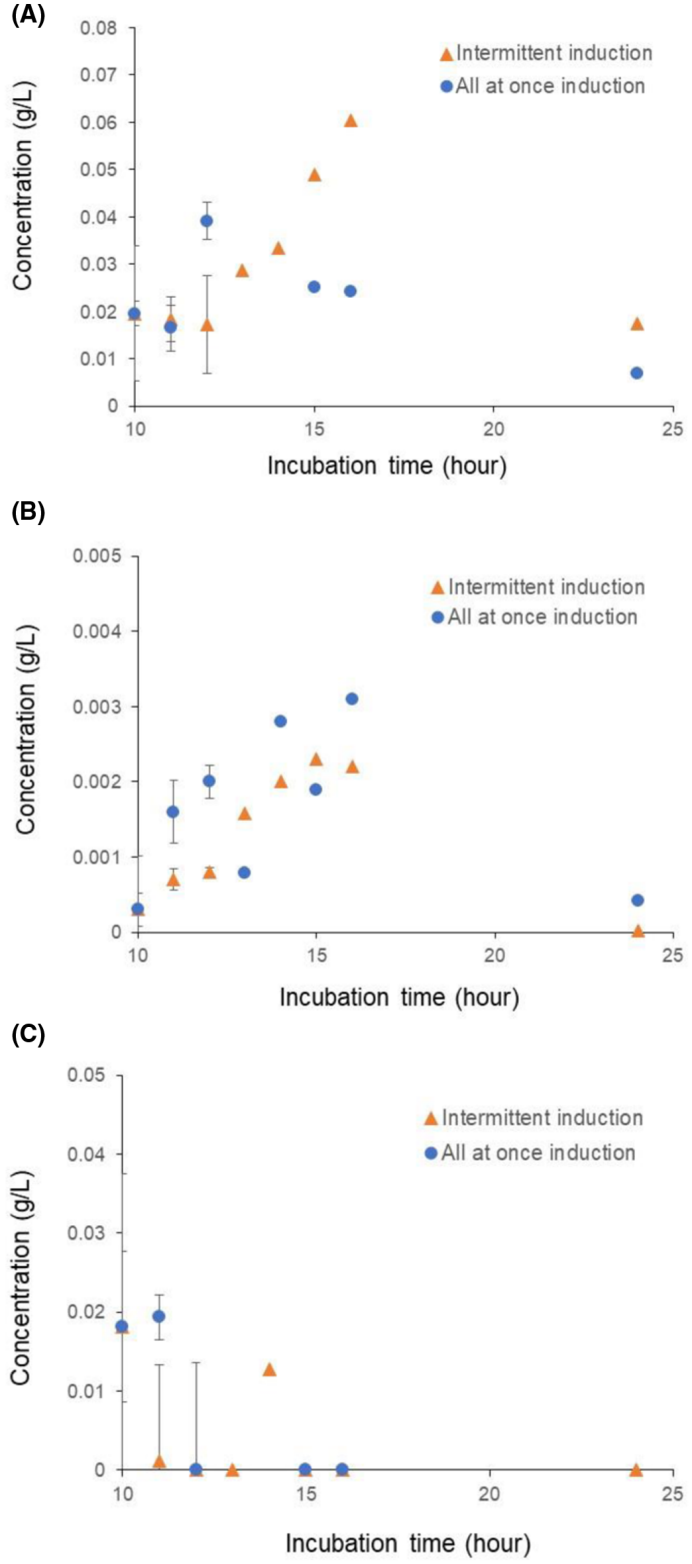
Concentration of lactose (A), glucose (B), and galactose (C) in culture media used for recombinant Bt, expressing the mutated LEA‐E peptide. The sample shows time dependence in intermittent (10 μL) and all‐at‐once (50 μL) induction by lactose. Induction time of intermittent (10 μL) was followed at the 11^th^, 12^th^, 13^th^, 14^th^, and 15^th^ h and all‐at‐once (50 μL) induction of lactose at the 11^th^ h. Data are mean values ± SD, with three independent repeats (*n* = 3). [Colour figure can be viewed at wileyonlinelibrary.com]

Comparing the data in Fig. 2A,B, at the 11^th^ h, Fig. 2A the lactose concentration was near about 0.02 g·L^−1^ both intermittent and all at once. Whereas in Fig. 2B the glucose concentration was lower than lactose both in intermittent (< 0.001 g·L^−1^) and all at once (< 0.002 g·L^−1^), indicating that as glucose sources get exhausted, the bacteria shift to using the second carbon source lactose that acts as an inducer, enhancing Cry protein expression. Another purpose of this experiment was to clarify which concentration and the pattern of induction (either intermittent or all‐at once‐induction) was more fruitful. From Fig. 2A, we can easily determine that the presence of lactose concentration (0.06 g·L^−1^) after intermittent induction was higher, whereas adding the entire volume of lactose all at once led to the lowering of the lactose concentration (> 0.03 g·L^−1^) at the 16^th^ h. Consequently, if we observe Fig. 2B, the concentration of glucose during an intermittent induction was lower (0.002 g·L^−1^) than it was in the case of all‐at‐once addition (< 0.03 g·L^−1^) of lactose at the 16^th^ h. Lactose was induced at the timepoint of the 11^th^ h where the samples were harvested after the 11^th^ h of incubation. Thus, Fig. 2B demonstrated the trend of the enhanced growth curve to digest the lactose by converting it to glucose and galactose. Figure 2C shows the absence of galactose at the 16^th^ h. For the 24^th^ h, the lactose concentration followed the same pattern as the 16^th^ h, where slowly it lowered both concentrations according to the time interval, but the intermittent concentration was higher than the all‐at‐once concentration. Induction of lactose till the 15^th^ h indicated that intermittent induction was more effective than all‐at‐once. Accordingly, the data of glucose concentration for the 24^th^ h followed the same pattern as the 16^th^ h, where the intermittent concentration of glucose was lower than the intermittent induction of lactose. Finally, we can conclude that the intermittent induction of lactose might be a new technique for the enhancement of bacterial protein expression.

Lactose, added intermittently for the induction of gene expression, enhanced the Cry protein expression in the LEA peptide coexpression system. Lactose‐induced culture better expressed the gene and can be used as an inducer, replacing the use of IPTG. The efficacy of lactose was checked by the intermittent induction to the media. This lactose‐induced system was not only more effective than IPTG, but also presented a new innovative technique of intermittent induction in comparison to the all‐at‐once induction. Intermittent induction allows the bacterial cell to properly perform the function of lactose for their growth and the lac operon system to enhance the expression of the LEA peptide and the protein of interest. Interestingly, the same amount of lactose was used in both the intermittent induction from the 11^th^ to the 15^th^ h and all‐at‐once induction (50 μL). The induction pattern (intermittent induction, 10 μL) enhanced the expression of the Cry protein three‐fold. Finally, the comprehensive utilization of intermittent induction of lactose in the near future may represent a new window to a more efficient expression of the protein in comparison to IPTG.

## Conflict of interest

The authors declare no conflict of interest.

## Author contributions

MA performed data curation, investigation, and methodology, performed formal analysis, wrote the original draft, and reviewed and edited the article. TS and MM provided resources, investigation, and methodology. YW performed investigation and formal analysis. TA, KM, and YI performed the investigation and methodology. SI conceptualized the study, contributed to funding acquisition and project administration, wrote the original draft, and reviewed, and edited the article.

## Data Availability

The data that support the findings of this study are available from the corresponding author [ikeno@life.kyutech.ac.jp] upon reasonable request.
